# Supplementary material for: Utility of Alternative Promoters for Foreign Gene Expression Using the Baculovirus Expression Vector System
Source: Viruses. 2022 Nov 29;14(12):2670. doi: 10.3390/v14122670 (PMC9786725; doi:10.3390/v14122670)
Supplement: Supplementary file 1 [file viruses-14-02670-s001.zip › viruses-1971328-supplementary.pdf]

Utility of alternative promoters for foreign gene expression using the baculovirus expression vector system: supplementary information

Mark R. Bruder and Marc G. Aucoin*

Department of Chemical Engineering, University of Waterloo, Waterloo, ON N2L 3G1, Canada

* Correspondence: maucoin@uwaterloo.ca; Tel.: +1-519-888-4567

The following contains: Figure S1: AcMNPV ORFs categorized according to transcript abundance; Figure S2: AcMNPV ORFs promoter motifs categorized according to transcript abundance; Figure S3: Sequences flanking the late gene promoter motif; Figure S4: Sequences flanking the translation initiation site; Table S1: Primers used in this study; Table S2: Promoters on commercially available BEVS transfer plasmids; Table S3: Position and sequence of putative upstream octamer matches in relation to TAAG motif; Table S4: Position and sequence of putative downstream octamer matches in relation to TAAG motif.

Figure S1

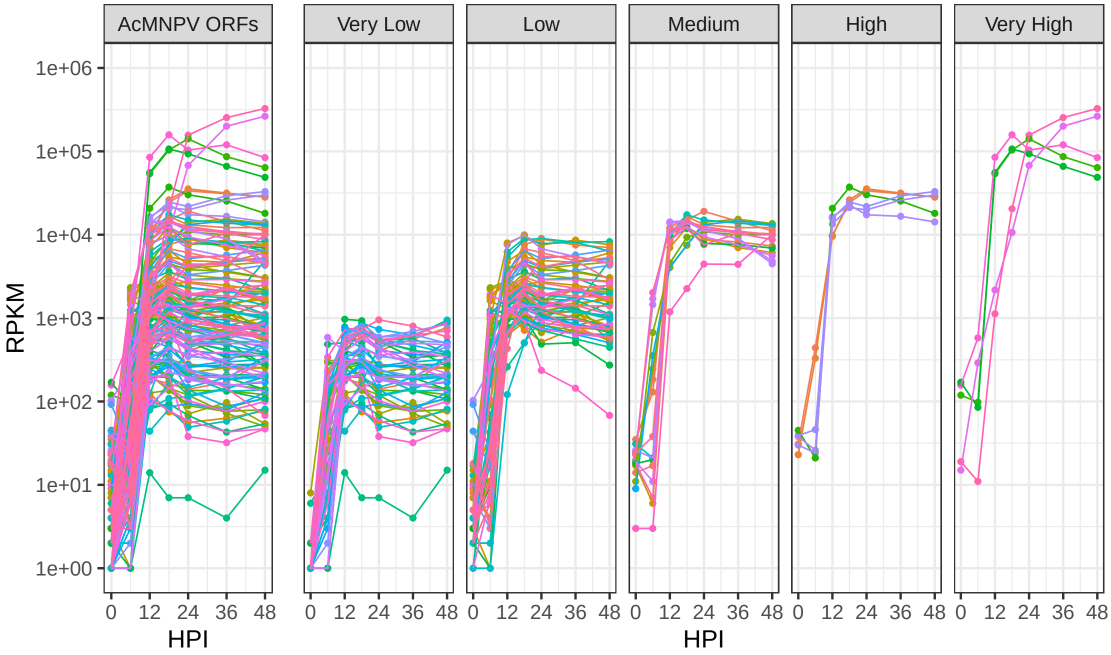

Figure S1: AcMNPV ORFs categorized according to transcript abundance.

Figure S2

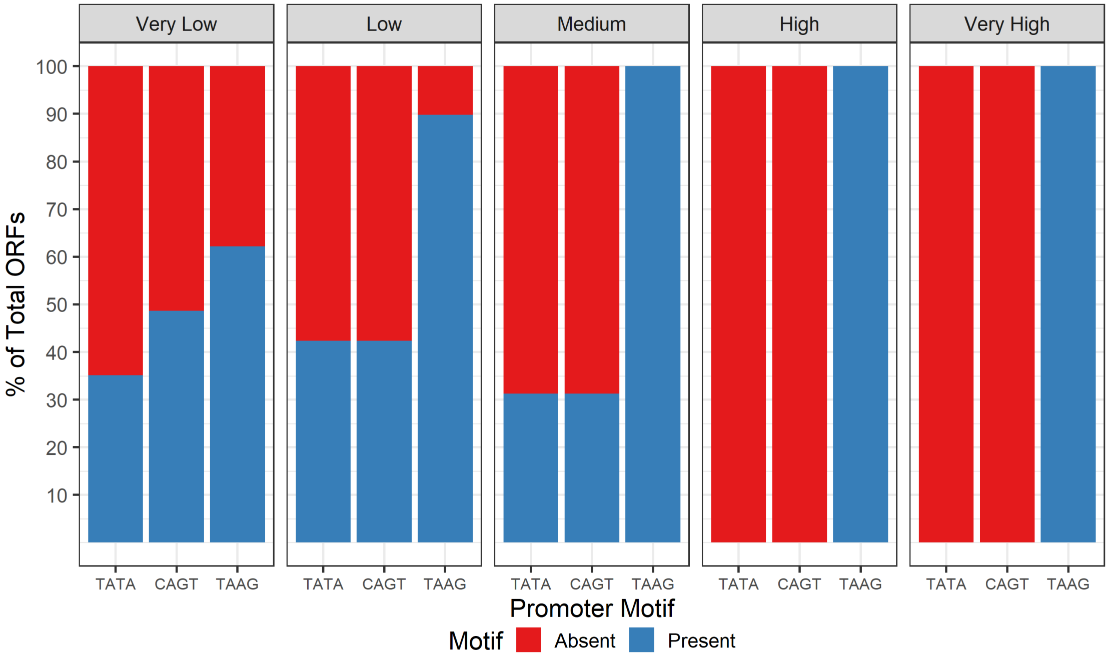

Figure S2: Proportion of AcMNPV ORFs with different promoter motifs, categorized according to transcript abundance. TATA and CAGT motifs are recognized and transcribed by the host RNAP II whereas the TAAG motif is recognized and transcribed by the viral RNAP.

Figure S3

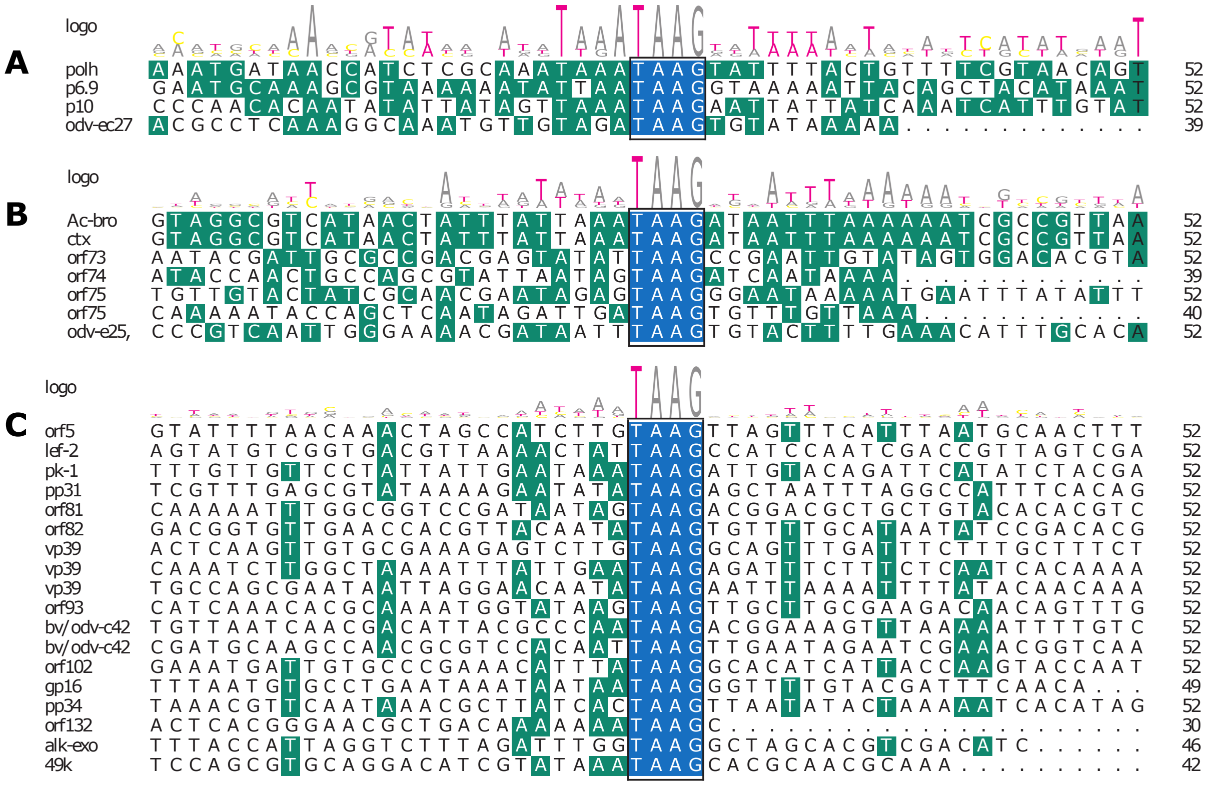

A
B
C
logo
polh
p6.9
p10
odv-ec27
Ac-bro
ctx
orf73
orf74
orf75
odv-e25,
orf5
lef-2
pk-1
pp31
orf81
orf82
vp39
orf93
bv/odv-c42
orf102
gp16
pp34
orf132
alk-exo
49k

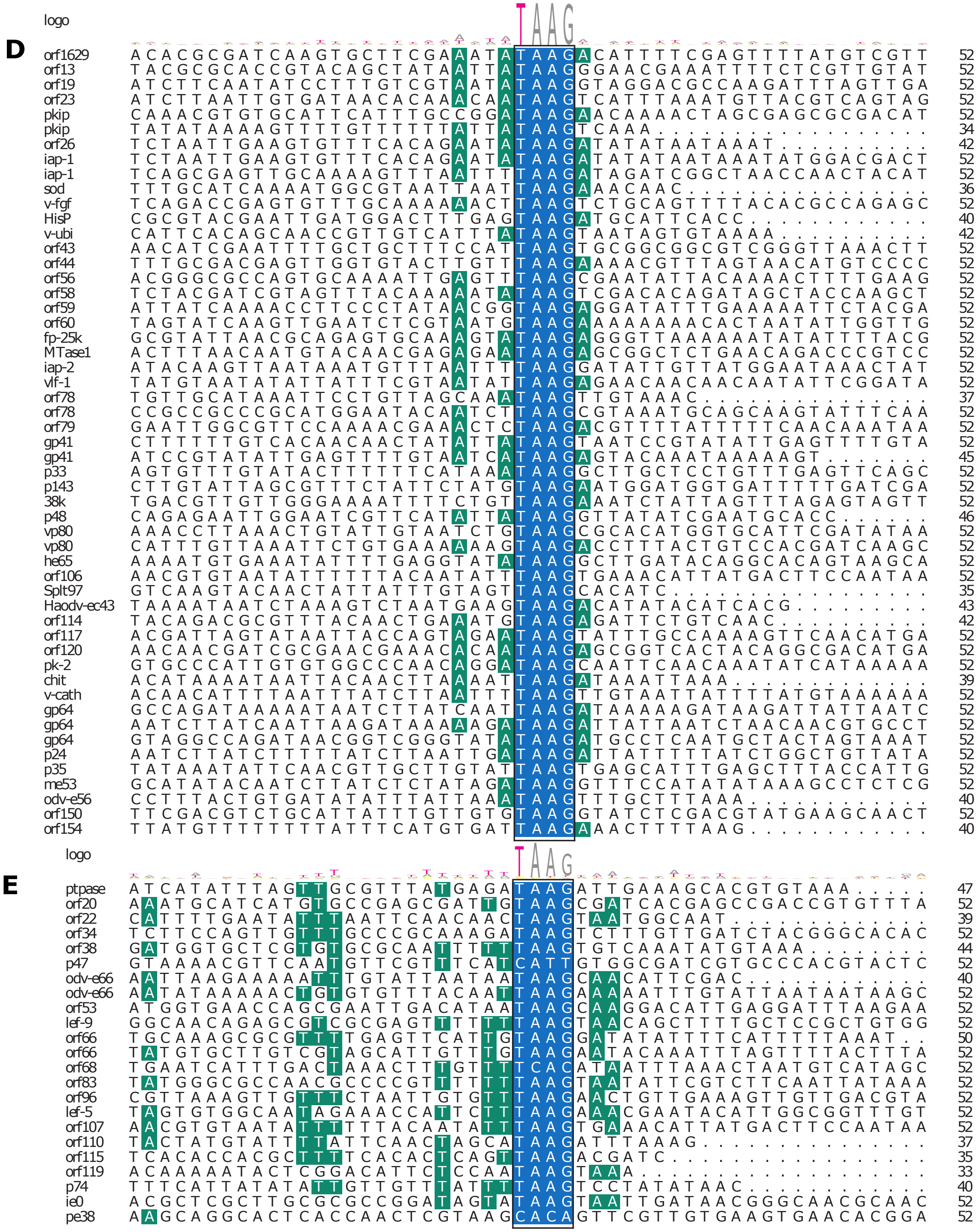

Figure S3: Sequences flanking the late gene promoter motif for A. Very High, B. High, C. Medium, D. Low, and E. Very Low classes.

Figure S4

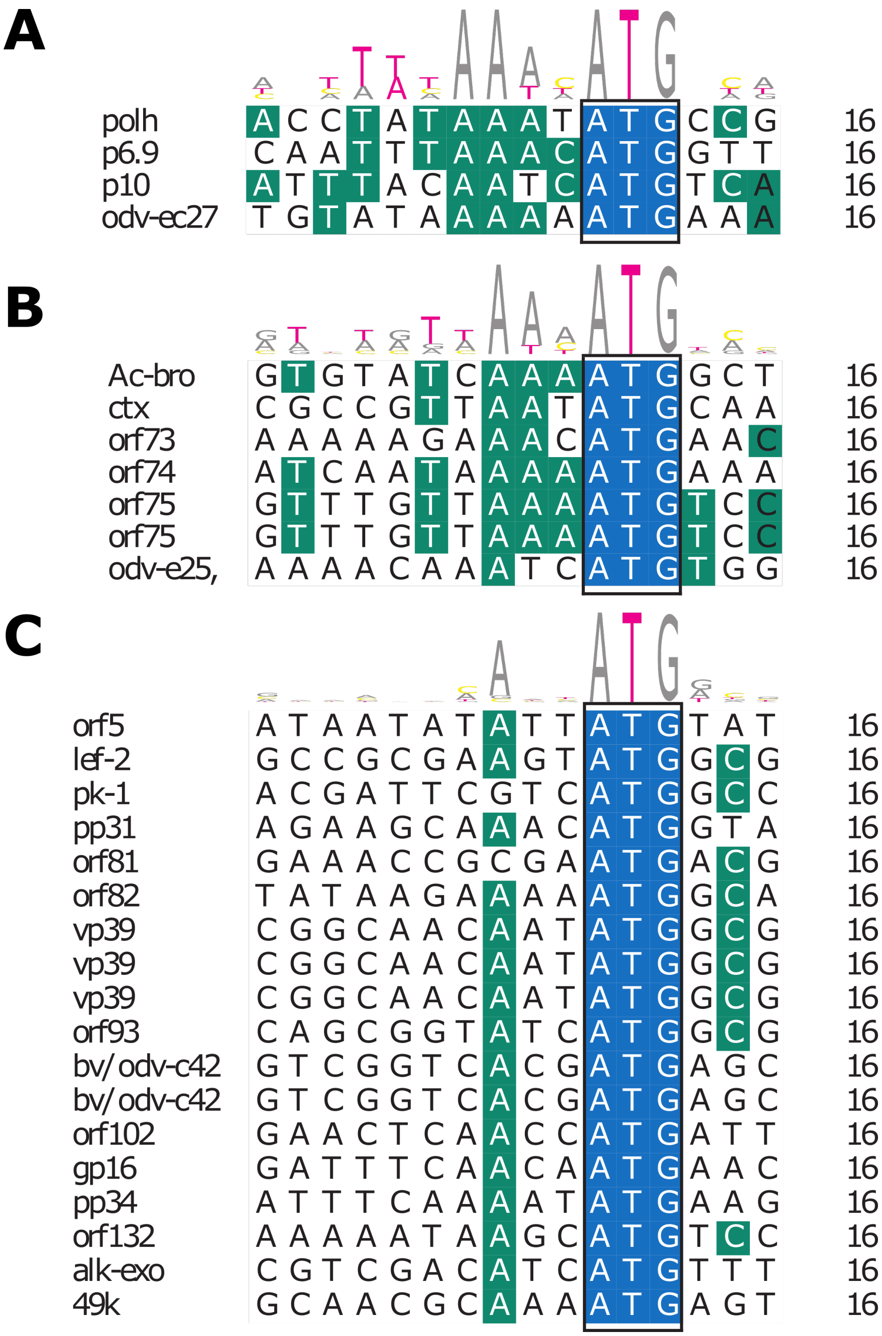
A
polh ACCTATAAATATGCCG 16
p6.9 CAATTTAAACATGGTT 16
p10 ATTTACAATCATGTCA 16
odv-ec27 TGTATAAAAAATGAAA 16
B
Ac-bro GTGTATCAAAATGGCT 16
ctx CGCCGTTAATATGCAA 16
orf73 AAAAAGAAACATGAAC 16
orf74 ATCAATAAAAATGAAA 16
orf75 GTTTGTTAAAATGTCC 16
orf75 GTTTGTTAAAATGTCC 16
odv-e25, AAAACAAATCATGTGG 16
C
orf5 ATAATATATTATGTAT 16
lef-2 GCCGCGAAGTATGGCG 16
pk-1 ACGATTCGTCATGGCC 16
pp31 AGAAGCAAACATGGTA 16
orf81 GAAACCGCGAATGACG 16
orf82 TATAAGAAAAATGGCA 16
vp39 CGGCAACAATATGGCG 16
vp39 CGGCAACAATATGGCG 16
vp39 CGGCAACAATATGGCG 16
orf93 CAGCGGTATCATGGCG 16
bv/odv-c42 GTCGGTCACGATGAGC 16
bv/odv-c42 GTCGGTCACGATGAGC 16
orf102 GAACTCAACCATGATT 16
gp16 GATTTCAACAATGAAC 16
pp34 ATTTCAAAATATGAAG 16
orf132 AAAAATAAGCATGTCC 16
alk-exo CGTCGACATCATGTTT 16
49k GCAACGCAAAATGAGT 16

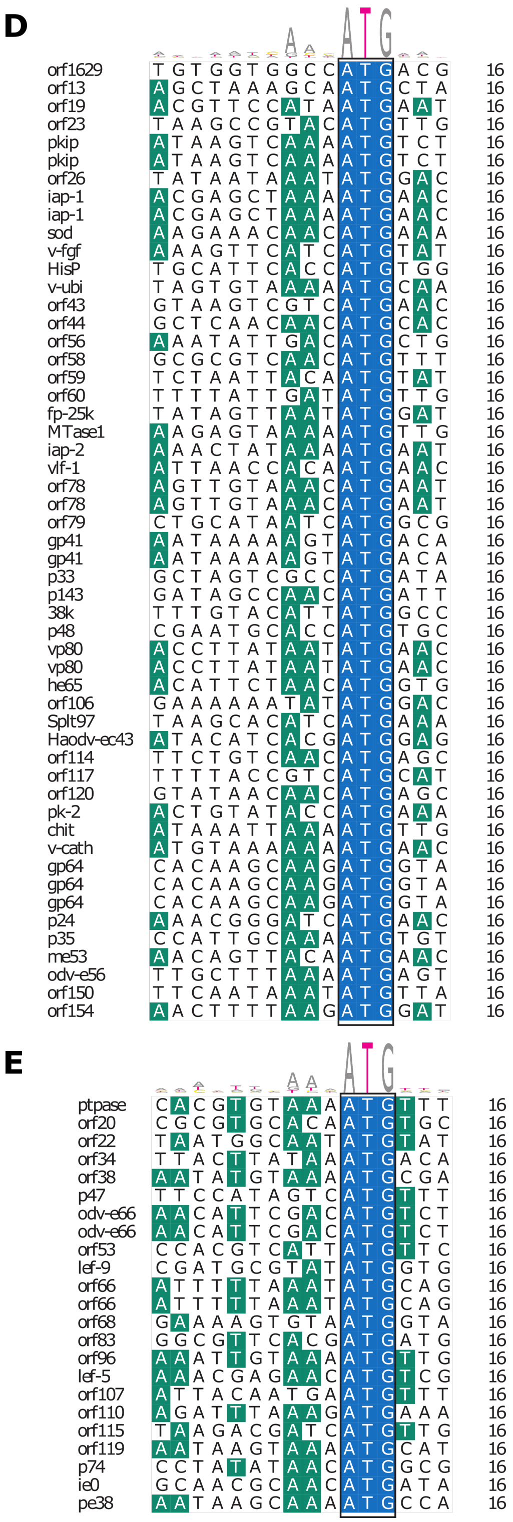

Figure S4: Sequences flanking the translation initiation site for A. Very High, B. High, C. Medium, D. Low, and E. Very Low classes.

Table S1: Primers used in this study

Plasmid Construct	Sequence (5'-3')	Description
Promoter-GFP	atggtgagtgtgattaagccc<br>cgctggactggcatgaac	pHR-GFP (promoterless)
	caccgaagttcatgccagtccagcgccacttgcgagttttgcag<br>cttcatctcgggcttaatcacactcaccat attgttgccgttataaatatgg	vp39 promoter
	caccgaagttcatgccagtccagcgcagcgaagaggagaacaaca<br>cttcatctcgggcttaatcacactcaccat tttaacaaacacttatcaatctattgagc	orf75 promoter
	caccgaagttcatgccagtccagcg tcgtacagctcagtgtacagtttg<br>gatcttcatctcgggcttaatcacactcaccat aatgtacaaaaatggaccagttacg	38k promoter
	caccgaagttcatgccagtccagcg ccccccaaaaaattgcac<br>gatcttcatctcgggcttaatcacactcaccat gtttgcttcttgtaaacctttgaaac	39k promoter
	caccgaagttcatgccagtccagcg tcgcccgacgatca<br>cttcatctcgggcttaatcacactcaccat attaacggcgatttttttaaattatc	ctx promoter
	caccgaagttcatgccagtccagcg ggttagttccagatagccatcg<br>cttcatctcgggcttaatcacactcaccat cttgcttgtgtgttccttattga	gp64 promoter
	gtatattaattaaaatac atggtgagtgtgattaagccc<br>ggcttaatcacactcaccat gtattttaattaatatacaaatgatttgataataattc	Δp10 promoter
	caccgaagttcatgccagtccagcg aaattccgttttgcgacg<br>catctcgggcttaatcacactcaccat gtttaaattgtgtaatttatgtagctgta	p6.9 promoter
	caccgaagttcatgccagtccagcgcgtaggcctttgaattccg<br>ggcttaatcacactcaccat atttataggtttttttattacaaaactgttacgaaaacag	polh promoter
Promoter-SEAP	ggcgcgccatgaatcgtttttaaaataac	pHR-promoter fwd
	atgcttctcttattgctgctgctgggcctgag<br>gttattttaaaaacgattcatggcgcgcc ttatgtctgctcgaagcggcc	SEAP gene
	caggcccagcagcagcaataagagaagcat attgttgccgttataaatatgg	vp39 rev
	caggcccagcagcagcaataagagaagcat tttaacaaacacttatcaatctattgagc	orf75 rev
	caggcccagcagcagcaataagagaagcat aatgtacaaaaatggaccagttacg	38k rev
	caggcccagcagcagcaataagagaagcat gtttgcttcttgtaaacctttgaaac	39k rev
	caggcccagcagcagcaataagagaagcat attaacggcgatttttttaaattatc	ctx rev
	caggcccagcagcagcaataagagaagcat cttgcttgtgtgttccttattga	gp64 rev
	caggcccagcagcagcaataagagaagcat gtattttaattaatatacaaatgatttg	Δp10 rev
	caggcccagcagcagcaataagagaagcat gtttaaattgtgtaatttatgtagctgta	p6.9 rev
	caggcccagcagcagcaataagagaagcat atttataggtttttttattacaaaactg	polh rev
qPCR primers	cgacgttgctttttgatcct<br>gcaacgacaagccatcagta	28S
	tctacgacatcaggttcgacgg<br>tccttcttggccttgtaggtgg	GFP
	agtacccagatgactacagc<br>ggatctcgtatttcatgtctcc	SEAP

Table S2: Promoters on commercially available BEVS transfer plasmids

System	Family	Plasmid	Expression	Promoter(s)	polyA
Transposition	pFastbac™	pFastbac-1, HT	single	polh	SV40
		pFastbac Dual	dual	polh/p10	SV40, HSV TK
	MultiBac™	pIDC, pIDK, pIDS	single‡‡	polh/p10	SV40, HSV TK
		pFL, pKL, pSPL, pUCDM	dual	polh/p10	SV40, HSV TK
		pACEBac1, pACEBac2	single‡‡	polh/p10	SV40, HSV TK
Homologous Recombination	pBAC™	pBAC-1/2/3	single	polh	n/a
		pBAC-4x	multi	polh/p10	synthetic
		pBAC-5	single	gp64	n/a
		pBAC-6†	single	gp64	n/a
		pBACsurf-1‡	single	polh	n/a
	pIEx/Bac™	pIEx/Bac-1/3/4/5	single	hr5 -ie1-p10	ie1
	pTriEx™	pTriEx-1.1/2/3/4/5/6/7	single	p10	rabbit β-globin
	pAB™	pAB-6xHis/GST/MBP	single	polh	n/a
		pAB-bee/bee-8xHis/bee-FH†	single	polh	n/a

†: secretion signal included; ‡: gp64 fusion for surface display; ‡‡:contains multiplication element for multigene compatability

Table S3: Position and sequence of putative upstream octamer matches in relation to TAAG motif.

ORF	Start	End	Sequence
polh	109	102	ATTGTAAT
p6.9	209	202	ATTACAAT
p6.9	152	145	ATTGCAAG
p6.9	131	124	ATTACAAT
p6.9	27	20	AATGCAAA
Ac-bro	203	196	ATTGCCAC
ctx	203	196	ATTGCCAC
orf73	120	113	ATTGAAAC
orf73	90	83	ATTGAAAA
orf73	34	27	ATTGCAAA
orf74	115	108	ATTGCATA
orf74	22	15	ACTGCCAG
orf74	7	0	ATAGTAAG
orf75	61	54	ATCGCAAT
orf75	19	12	ATCGCAAC
orf75	135	128	ATTATAAG
odv-e25	96	89	ATTGCGAA
orf5	185	178	TTTGCATG
orf5	181	174	CATGCAAG
orf5	160	153	ATTGCGAT
orf5	7	0	CTTGTAAG

Continued on next page

Table S3 – continued from previous page

ORF	Start	End	Sequence
lef-2	115	108	ATTGTAAT
pk-1	153	146	TTGGCAAG
pp31	222	215	ATTGCAGG
pp31	165	158	ATTGCACG
pp31	116	109	AATACAAG
orf81	89	82	AATGCAAT
orf81	36	29	ATTTCAAA
orf81	7	0	ATAGTAAG
orf82	200	193	ATTTCATG
orf82	75	68	ATTTCAAT
vp39	203	196	CTTGCGAG
vp39	72	65	ATTTCAAT
vp39	7	0	CTTGTAAG
vp39	145	138	ATTGCAAG
vp39	223	216	CTTGTAAG
vp39	97	90	ATTGCAAG
orf93	215	208	AGTGCATG
orf93	110	103	GTTGCAAG
bv/odv-c42	150	143	GTCGCAAG
bv/odv-c42	72	65	GTTGCAAA
bv/odv-c42	159	152	GTTGCAAA
bv/odv-c42	27	20	GATGCAAG
orf102	71	64	ATTGAAAT
gp16	170	163	ATAGCAAC

Continued on next page

Table S3 – continued from previous page

ORF	Start	End	Sequence
gp16	124	117	GTTGCAAG
pp34	147	140	CTTGCAAA
pp34	103	96	ATGGCAAA
orf132	142	135	TTTGCTAG
alk-exo	52	45	AATGCAAT
alk-exo	46	39	ATTGGAAC
49k	155	148	AATGCAAT

Table S4: Position and sequence of putative downstream octamer matches in relation to TAAG motif.

ORF	Start	End	Sequence
p6.9	99	92	ATTAGGAA
p6.9	47	40	ATTTGGGA
p6.9	8	1	ATTAATAA
p10	105	98	ATTCAGAA
p10	72	65	ACTATGAA
p10	64	57	ATTATGCA
odv-ec27	170	163	AGTAGTAA
odv-ec27	37	30	TTTATGAA
orf73	141	134	TTTAGAAA
orf73	100	93	ATAAGGAC
orf74	208	201	AGTAGAAA
orf74	64	57	TTTAGCAA
orf74	8	1	AATAGTAA
orf75	203	196	AAAAGGAA
orf75	132	125	ATAAGTAA
orf75	129	122	AGTAAGAA
odv-e25	160	153	ATTATGTA
odv-e25	119	112	GTTCGGAA
odv-e25	96	89	ATTGCGAA
odv-e25	21	14	ATTGGGAA
lef-2	102	95	TTTACGAA

Continued on next page

Table S4 – continued from previous page

ORF	Start	End	Sequence
pp31	141	134	ATTCGGAC
orf81	209	202	ATTATCAA
orf81	8	1	AATAGTAA
orf82	90	83	TTTAAGAA
vp39	182	175	GTTTGGAA
vp39	130	123	AATAGGTA
vp39	64	57	ATTAGGAA
vp39	134	127	GTTTGGAA
vp39	82	75	AATAGGTA
vp39	16	9	ATTAGGAA
orf93	8	1	ATAAGTAA
orf102	147	140	AGTTGGAA
orf102	115	108	ATGAGCAA
orf102	49	42	ATTTGTAA
gp16	183	176	ATTTTGAA
gp16	132	125	ATTAACAA
pp34	60	53	ATTAACAA
alk-exo	143	136	ATCAAGAA
alk-exo	136	129	ACTAAGAA
alk-exo	92	85	GTTGGGAA
alk-exo	47	40	AATTGGAA
alk-exo	22	15	ATTAGGTC
alk-exo	9	2	ATTTGGTA
49k	210	203	ATTAATAA

Continued on next page

Table S4 – continued from previous page

ORF	Start	End	Sequence
49k	192	185	GTTATGAA
49k	60	53	ATGTGGAA
